# Integrative Hedonic and Homeostatic Food Intake Regulation by the Central Nervous System: Insights from Neuroimaging

**DOI:** 10.3390/brainsci12040431

**Published:** 2022-03-24

**Authors:** Alejandro Campos, John D. Port, Andres Acosta

**Affiliations:** 1 Precision Medicine for Obesity Program, Division of Gastroenterology and Hepatology, Department of Medicine, Mayo Clinic, Rochester, MN 55905, USA; campos.alejandro@mayo.edu; 2 Department of Diagnostic Radiology, Mayo Clinic, Rochester, MN 55905, USA; port.john@mayo.edu

**Keywords:** food intake regulation, reward, fMRI, hedonic eating, gut–brain axis, neuroimaging

## Abstract

Food intake regulation in humans is a complex process controlled by the dynamic interaction of homeostatic and hedonic systems. Homeostatic regulation is controlled by appetitive signals from the gut, adipose tissue, and the vagus nerve, while conscious and unconscious reward processes orchestrate hedonic regulation. On the one hand, sight, smell, taste, and texture perception deliver potent food-related feedback to the central nervous system (CNS) and influence brain areas related to food reward. On the other hand, macronutrient composition stimulates the release of appetite signals from the gut, which are translated in the CNS into unconscious reward processes. This multi-level regulation process of food intake shapes and regulates human ingestive behavior. Identifying the interface between hormones, neurotransmitters, and brain areas is critical to advance our understanding of conditions like obesity and develop better therapeutical interventions. Neuroimaging studies allow us to take a glance into the central nervous system (CNS) while these processes take place. This review focuses on the available neuroimaging evidence to describe this interaction between the homeostatic and hedonic components in human food intake regulation.

## 1. Introduction

Energy balance in humans is a complex process. Its regulation is synchronized by metabolic status and strongly influenced by reward circuitries within the central nervous system (CNS). Food intake is divided into hunger, satiation, and satiety. Hunger can be defined as the drive to consume. On the other hand, satiation is the process that brings an eating episode to an end (intra-meal inhibition). Satiety is the process that inhibits eating in the postprandial period (inter-meal inhibition) [1]. These dynamic stages are controlled by the homeostatic and hedonic components of food intake regulation.

**Figure 1:**
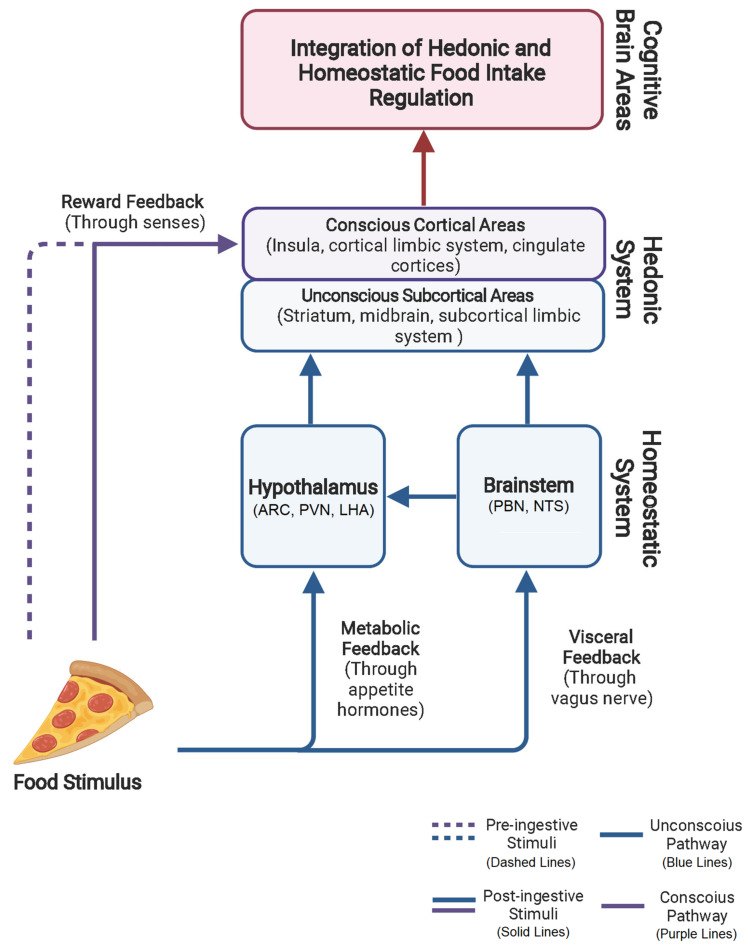
Graphical Abstract. Integration of the hedonic and homeostatic components of food intake regulation. Food stimuli are perceived during a pre-ingestive (dashed lines, purple or blue) phase by the sense of sight, smell, audition, and touch. After food ingestion, sensorial information from food’s taste, smell, consistency, and temperature constitutes a second stimulus inside the oral cavity (post-ingestive stimuli (solid lines, purple or blue)). This information travels to the central nervous system (CNS) through a “fast” and conscious pathway. At this point, the energetic and macronutrient content of food has not been sensed. However, once the food arrives at the gut lumen, it stimulates the release of gut hormones, such as peptide Y Y (PYY), glucagon-like peptide 1 (GLP-1), cholecystokinin (CCK), insulin, and others, based on the macronutrient composition of the food ingested. This peripheral metabolic feedback travels to the CNS as a signal of energy intake. Additionally, the mechanic distention of the gut is sensed by vagal afferents and projected to the brainstem to inform about the sensation of fullness. The unconscious metabolic and visceral feedback (bottom blue lines and boxes) and the conscious reward feedback (left dashed and solid purple lines) are integrated within brain areas associated with aversion, cognition, reward, motivation, memory, and decision making (middle blue and purple boxes). From these areas, information projects to higher cognitive brain centers to ultimately regulate eating behavior (top red lines and boxes). ARC, arcuate nucleus; PVN, paraventricular nucleus; LHA, lateral hypothalamic area; PBN, parabrachial nucleus; NTS, nucleus tractus solitarius/nucleus of the solitary tract.

Food intake is regulated by different organs and systems that project feedback of the individual’s metabolic status to the CNS, which ultimately controls this process (Figure 1). The initiation, duration (termination of a meal), and timing (timing between meals) of eating episodes depend on conscious and unconscious neurohormonal processes, some from the internal milieu and others coming from the surrounding environment and decoded by the senses (e.g., sight, smell, taste). This regulation process depends on two different but closely intertwined processes: the homeostatic (i.e., metabolic) and hedonic (i.e., reward) components of food intake.

Homeostatic regulation of food intake is mainly controlled by the sensing of metabolic and visceral feedback by the hypothalamus [2]. In the CNS, the hypothalamus receives metabolic information from hormones and peptides released from the gut in response to food and its macronutrient composition, adipose tissue signals, and visceroceptive information from the vagus nerve. This homeostatic feedback from the gut, adipose tissue, and the vagus nerve is processed and decoded into onset or offset eating signals mainly by the leptin-melanocortin pathway in the arcuate and paraventricular nuclei. This metabolic sensing by the hypothalamus is entirely unconscious and coordinates hunger, satiety, and satiation by strongly modulating the intra- and inter-meal rewarding properties of food, a process defined as appetition [3,4].

Hedonic eating is the process that drives the individual to eat solely to elicit pleasurable feelings and/or to escape from anhedonic states, disregarding the individual’s metabolic status or the nutritional value of the food consumed [5]. The hedonic component of food intake relies on conscious sensorial information from food’s characteristics that provide subjective liking and an unconscious process modulated by appetite gut hormones. This metabolic feedback enhances and reinforces the salience of foods. De Araujo et al. proposed that the process of hedonic eating consists of a flavor-nutrient (conscious–unconscious) paradigm, and the insula has been proposed as the converging structure for this conscious and unconscious processing [6]. As previously mentioned, this concept has been previously explored by Scalfani, defined as appetition, which is a post-oral process where nutrient properties of food promote intake beyond inhibition processes (e.g., satiety and satiation) [3].

Most of our current knowledge on the neural control of food intake regulation comes from experiments in rodents and neuroimaging studies in humans [7]. Research techniques such as surgical dissection, gene knockout, designer receptors exclusively activated by designer drugs, and opto- and chemo-genetics [8,9,10,11] cannot be performed in humans and, thus, need a proxy to be studied in humans. Neuroimaging techniques such as functional magnetic resonance imaging (fMRI), positron emitted tomography (PET), functional near-infrared spectroscopy [12], and single-photon emission computed tomography (SPECT) have become the backbone of food intake regulation study in humans [13,14]. fMRI and perfusion MRI techniques are the most widely used neuroimaging tools to study food intake regulation in humans. They indirectly assess brain activity by measuring the change in cerebral blood flow (CBF), cerebral blood volume, and/or blood oxygenation. Blood oxygen level-dependent (BOLD) fMRI and artery spin labeling (ASL) MRI (and its proton labeling technique variants (CASL, PASL, pCASL)) are two widely used techniques in neuroimaging [15]. BOLD-fMRI measures the level of deoxygenated hemoglobin, while ASL-MRI—and its variations—measures brain perfusion. Both these techniques attempt to assess neural activity through changes in their respective measurements (e.g., blood flow, oxygenation, or volume). However, they are still an indirect proxy of neural activity [16]. In general, neuroimaging studies food intake regulation through the brain responses to visual or ingestive food stimuli. Interestingly, some neuroimaging studies have also demonstrated that physical activity and exercise—key elements in energy balance regulation—play an important role in the neural control of food intake [17,18,19,20]; however, this topic goes beyond the scope of this study.

This review will focus on the current neuroimaging evidence in humans to understand how the CNS integrates homeostatic and hedonic signals to regulate food intake.

## 2. Methods

We searched PubMed, Scopus, MEDLINE, and Google Scholar databases for neuroimaging studies (BOLD-MRI, ASL, CASL, PASL, pCASL, PET, SPECT) in humans from 1 January 1990 to 1 January 2021, with no language restrictions, assessing brain responses to food stimuli (visual or ingestive) and/or administration or measurement of gut hormones, mechanical stimulus (gastric distention), or after pharmacological intervention. We used a range of terms, including “MRI, fMRI, PET, SPECT, ASL, CASL, PASL, pCASL, neuroimaging, neural, brain response(s), homeostatic, hedonic, reward, hormonal, peptide YY (PYY), glucagon-like peptide 1 (GLP-1), cholecystokinin (CCK), and ghrelin, leptin, dopamine (DA), serotonin (5-HT)”. We focused on neuroimaging studies that were relevant to this review’s approach to how the CNS integrates homeostatic and hedonic signals. We incorporated some animal studies to provide a comprehensive background and support findings from the cited neuroimaging studies.

## 3. Homeostatic Food Intake Regulation

A case report in 1901 was perhaps the first to distinguish the CNS in the regulation of energy homeostasis when the author observed how one of his pediatric patients developed obesity as a consequence of a tumor involving the diencephalon [21,22]. Approximately fifty years after Fröhlich’s report, Anand demonstrated that ablative lesions in the ventromedial nucleus (VML) of rodents led to voracious eating and obesity; electrical stimulation of this area led to starvation and death [11]. These observations confirmed the role of the hypothalamus as a regulatory feeding center. Despite identifying the structure that regulated food intake, the signals in this regulation were still unknown. Decades later, in a sophisticated parabiosis experiment, Hervey connected the circulation of two rodents and created ablative lesions in the VML of one of them; this caused the animal to overeat and gain weight; interestingly, the other connected rodent (with no ablative lesions) decreased its food intake, causing it to lose weight. These observations led him to hypothesize that suppression in food intake was caused by adiposity-related circulating factors [23]. In 1994, Zahn and colleagues discovered the adipose-derived hormone leptin, unveiling another piece in the energy regulation puzzle [24,25]. In the past years, the discovery of monogenic obesity-related mutations has unveiled more information about how the brain regulates energy balance, especially food intake [26,27,28]. Interestingly, most genes associated with obesity are expressed in the CNS, particularly in the hypothalamus and brain areas associated with reward, memory, and decision making [29].

### 3.1. Peripheral Metabolic Signals

Homeostatic regulation depends mainly on the release of hormones and peptides and visceral information from the gastrointestinal tract. Gut hormones such as PYY, GLP-1, CCK, and ghrelin are secreted in response to macronutrients from food arriving in the gut lumen [30,31]. These signals travel through the bloodstream to reach the CNS, where they can act as neurotransmitters and are decoded into short-term onset or offset eating signals [32,33,34,35,36,37,38]. Leptin plays a crucial role in regulating energy homeostasis [25], acting as a satiety signal when present but strongly driving hunger when absent, decreased, or non-properly sensed [25,39]. The vagus nerve carries visceral information from the gut to the brainstem, where it is processed and projected to the hypothalamus (and other brain areas) [40].

#### 3.1.1. Leptin

Leptin is secreted from adipocytes in the white adipose tissue and is directly correlated with the degree of adiposity, with the hypothalamus being its primary site of action in the CNS [41]. Leptin’s primary role in the energy balance is to increase hunger in response to decreased or absent leptin levels in the bloodstream. The CNS interprets this leptin-deficient status as an energy deficit state, triggering counteracting responses like increased appetite and reduced resting energy expenditure to compensate for the energy-deficient state [42]. Interestingly, an acute decrease in leptin levels has been observed after 48 h caloric restriction, suggesting an acute regulation of energy balance by leptin without changes in weight or adiposity [43]. Leptin accesses the CNS through the median eminence, reaching the third ventricle, and is distributed across the CNS through the ventricular system [44]. Leptin activates pro-opiomelanocortin/cocaine- and amphetamine-regulated transcript (POMC/CART), expressing neurons in the arcuate nucleus of the hypothalamus (ARC) through an increase in c-Fos concentrations, and inhibits the Agouti-related peptide/neuropeptide Y (AgRP/NPY), expressing neurons via an increase in the suppressor of cytokine signaling 3 expression [45,46,47].

Most neuroimaging studies involving leptin have been carried out in leptin-deficient patients. Farooqi et al. demonstrated the crucial role of leptin in energy balance regulation by the brain using fMRI by showing that children with congenital leptin deficiency had increased activity in striatal brain areas such as the nucleus accumbens (NAc), putamen, amygdala, and hippocampus when presented with food cues. After seven days of leptin replacement therapy, the activity in these regions reduced dramatically, suggesting that leptin decreases hunger by altering striatal activity and the rewarding properties of food [48]. A similar study in leptin-deficient adults showed decreased activation of visual food cues in the insula and the parietal and temporal cortices and increased activation in the prefrontal cortex (PFC) [49]. Another study in leptin-deficient patients showed an altered response to visual food cues in the posterior cerebellum after leptin administration [50]. The cerebellum has been linked with detecting blood-borne nutritional signals and food anticipation in rodents [51]. The latter findings suggest that the cerebellum might play a role in the homeostatic regulation of food intake. Grosshans et al. observed an association between leptin levels and the activity in the ventral striatum [52]. Using BOLD-fMRI, Rosenbaum et al. observed that individuals in a weight-reduced state displayed increased activity in emotional, executive, and sensorial brain areas that were reversible by leptin replacement during the weight loss phase, similar to the observations in leptin-deficient patients observed by Farooqi et al. [48,53]. These findings suggest that the brain might interpret the weight-reduced state as similar to a leptin-deficient one, increasing responsiveness to food cues and predisposing the individual to an increased caloric intake.

#### 3.1.2. Ghrelin

Ghrelin is an orexigenic 28 amino acid peptide secreted from oxyntic glands in the gastric fundus and the ARC [54,55]. Ghrelin is secreted before a meal, influenced by cephalic-phase reflexes, weight loss, fasting, and insulin-induced hypoglycemia. It is catalyzed by ghrelin O-acyltransferase to its active form, acyl-ghrelin; despite its strong effect on food intake, acyl-ghrelin constitutes only around 10% of total circulating ghrelin, with a half-life of about 30 min [56]. In the CNS, ghrelin receptors are expressed in AgRP/NPY expressing neurons in the ARC, where it stimulates the release of AgRP and NPY to inhibit melanocortin receptor 4 (MC4R), expressing neurons in the paraventricular nucleus (PVN), increasing food intake, and antagonizing the effects of POMC neurons [57]. Recent fMRI studies have demonstrated that ghrelin influences reward-related brain areas, suggesting that it drives food intake by increasing the rewarding properties of food. Food reward is essential in decision making, the motivational drive to feed, and the physical work required to obtain energy resources. When animals are injected with ghrelin into the ventral tegmental area (VTA), DA activity increases in the NAc and motivates the animal to work harder to obtain the food-related reward [58].

Additionally, the growth hormone secretagogue receptor 1a has been implicated in memory and learning processes in mice [59]. In an fMRI study in human volunteers, ghrelin injection showed an increased reward prediction error (i.e., the difference between the received and the predicted reward) signaling in DA structures and enhanced food odor conditioning, remarking the importance of ghrelin as a link between the homeostatic and hedonic systems in food intake regulation [60]. Ghrelin also increases hunger sensations and the rewarding properties of food similar to a fasting state [61,62,63]. Additionally, ghrelin suppresses nutrient-dependent activation of the hypothalamus and limbic areas [64]. In an exciting experiment studying the FTO gene, subjects with the AA (high risk) allele demonstrated higher ghrelin levels and increased fMRI activity to visual food cues in homeostatic and reward brain areas compared with subjects carrying the TT (low risk) allele [65]. This finding could explain how weight gain associated with the FTO gene might be related to underlying higher ghrelin levels and a consequent increase in hunger and food-related reward.

#### 3.1.3. Cholecystokinin

CCK is secreted from I-enteroendocrine cells (EECs) located primarily in the duodenum and proximal jejunum. Its secretion is mainly stimulated by fat content in a meal. CCK receptors are present in vagal afferent neurons, cerebral cortex, thalamus, hypothalamus, basal ganglia, and dorsal hindbrain, where it acts as a neurotransmitter [66]. CCK has been shown to reduce meal size and specifically signal satiation. Moreover, elevated ingestion of a high-fat meal significantly increases CCK levels, which are negatively correlated with the activity in reward- and taste-related brain areas [67]. Furthermore, CNS structures such as the motor cortex and the anterior and posterior cerebellum have also been demonstrated to have CCK-dependent activation after a high-fat load [68].

#### 3.1.4. Glucagon-like Peptide-1

Formed from the cleavage of the preproglucagon, GLP-1 is secreted by L-EECs in the ileum and colon in response to luminal nutrients [69] but is also secreted from a small subset of neurons in the nucleus of the solitary tract (NST) [70]. GLP-1 increases glucose-dependent insulin release, decreases glucagon secretion, and delays gastric emptying. It also reduces food intake via GLP-1 receptors in the ARC, PVN, NST, and area postrema (AP) [71]. GLP-1 is degraded by the enzyme dipeptidyl-peptidase-4 (DPP-4), a proline/alanine-specific peptidase found on the luminal surface of capillary endothelial cells and the liver and blood vessels [72].

The anorexigenic effects of GLP-1 appear to be related to its influence on brain areas that regulate the hedonic properties of food [73]. GLP-1 analogs have been shown to play an essential role in conditioned taste avoidance, most likely by inducing aversive gastrointestinal symptoms such as nausea and vomiting [74,75,76]. In an fMRI study, administration of individual PYY and GLP-1 and a combined infusion were studied against placebo. Activation of reward brain areas (i.e., amygdala, insula, caudate, nucleus accumbens, orbitofrontal cortex [OFC], and putamen) to food cues was decreased after GLP-1 administration. The latter effect was additive when the combined (GLP1 + PYY) infusion was administered; the effects of both individual and combined infusions were similar to those observed in fMRI scans after meal ingestion [77]. Interestingly, another study showed higher hypothalamic connectedness in fMRI scans in GLP-1-responsive patients who had an anorexigenic effect (i.e., >10% decrease in energy intake) after the administration of exenatide, a GLP-1 analog [78]. In another study in patients with type 2 diabetes, consumption of a 450 cal (1883 kJ) meal reduced bilateral fMRI activity to visual food cues in the insula; interestingly, this reduction was not observed after a blockade of endogenous GLP-1 activity with GLP-1 receptor antagonist exendin 9–39 [79]. Farr and colleagues demonstrated the presence of GLP-1R in the hypothalamus, medulla, and parietal cortex through immunohistochemistry in human brain autopsies; then, in another randomized cohort of patients with type 2 diabetes, there was observed decreased fMRI activity in the parietal cortex, insula, and putamen after 17 days of treatment with liraglutide compared with placebo [80]. As mentioned earlier, the insula has been proposed as the brain structure where internal (unconscious) and external (conscious and unconscious) food-related signals converge [6].

#### 3.1.5. Peptide Y (PYY)

The hormone PYY is secreted from L- EECs in the ileum and colon in response to a meal’s fat content, especially short-chain fatty acids [81]. Circulating PYY is a combination of its secreted form PYY_1-36_ and its active form PYY_3-36_, which results from the cleavage of the NH2 terminal by DPP-4 [82,83]. During fasting, PYY_1-36_ levels predominate; however, after meal initiation, this is reversed, and PYY_3-36_ rapidly becomes the dominant subform in the bloodstream [84]. PYY_3-36_ has anorexigenic effects across multiple CNS structures, predominantly on circumventricular organs and Y2 receptors on vagal afferents [85]. Interestingly, PYY appears to cross the blood–brain barrier (BBB), inhibiting NPY receptors (YR) in the hypothalamus [82]. As mentioned earlier, PYY decreased the activation of visual food cues in reward-related brain regions when administered alone, and the effect was more significant when combined with GLP-1 [77]. Batterham et al. observed an increased activation after PYY_3-36_ infusion in the insula, anterior cingulate cortex (ACC), and ventral striatum and in the frontal, parietal, temporal, and cerebellar cortices. Interestingly, the degree of activation observed in the OFC after PYY_3-36_ administration correlated negatively with meal pleasantness perception [86].

A myriad of other gut hormones such as gastric inhibitory polypeptide, glicentin, and oxyntomodulin has also been associated with modulation of brain reward areas [87]; however, their discussion is beyond the scope of this review [88].

In summary, gut hormones are secreted in response to the macronutrient composition of food and act as short-term orexigenic and anorexigenic signals in the hypothalamus. The long-term signal leptin seems to modulate these short-term signals by enhancing their satiety/satiation effects and dampening hunger sensation when present but exerting the opposite effects (increased hunger and decreased satiety/satiation) when decreased or absent. Together, this homeostatic metabolic feedback is sent from the hypothalamus to key brain areas that regulate food intake.

### 3.2. Visceroceptive Feedback

Visceral information from the gut is carried by the vagus nerve (X). This information is key in the homeostatic regulation of food intake. Efferents from the vagus nerve pace the rate of the nutrient absorption, storage, and mobilization of food by influencing motility and secretion throughout the alimentary tract. Conversely, vagal mechanoreceptive afferents in the gastric mucosa detect the presence of ingested foods, while afferents in the external muscle layer, sensitive to stretch and tension, provide information about the volume of ingested foods [40]. Interestingly, the activity of taste-processing neurons in the visceral portion of the somatosensory cortex receives gastric distention feedback from the vagus nerve. This remarks a possible mechanism by which fullness modulates taste perception [89]. Feedback from the vagus nerve informs homeostatic and hedonic brain areas about food characteristics and aid meal size, initiation, timing, and termination. One study explored the neural correlates of gastric distention using either gradual intragastric balloon inflation (IGB) or intragastric nutrient infusion using H_2_^15^O PET [90]. Interestingly, subjects perceived the IGB distension to be more painful than the distention induced by the nutrient drink infusion, despite that both distension techniques used the same volume. More importantly, the nutrient-distention generated a progressive deactivation in pain-related areas such as the insula, ventrolateral PFC, ventromedial PFC (vmPFC), and somatosensory cortex, while IGB-distention generated progressive activation of these pain-related areas. Additionally, only nutrient-distension generated midbrain activation and significantly decreased and increased ghrelin and PYY_3-36_, respectively. These findings remark the importance of gut hormones in modulating visceral pain related to food consumption. Stephan and colleagues identified increased CBF in H_2_^15^O PET scans in the dorsal brainstem, left inferior frontal gyrus, insular cortex, and ACC after proximal IGB distension [91]. In a study with IGB and BOLD-fMRI in healthy subjects, Wang and colleagues observed activation in the sensorimotor cortices and insula with IGB volume <250 mL, in the left posterior amygdala and left posterior insula with 250–500 mL, and in the left precuneus with >500 mL [92].

Together these observations suggest that the visceroceptive information from vagal afferents regulates food intake by activating brain areas associated with sensorimotor functions, taste, pain, and aversion. Moreover, the anorexigenic effect of the mechanical stimulus produced by food is enhanced by gut hormones secreted in response to the macronutrient composition of food.

### 3.3. Central Nervous System Regulation

#### 3.3.1. The Hypothalamic Leptin–Melanocortin Pathway

**Figure 2:**
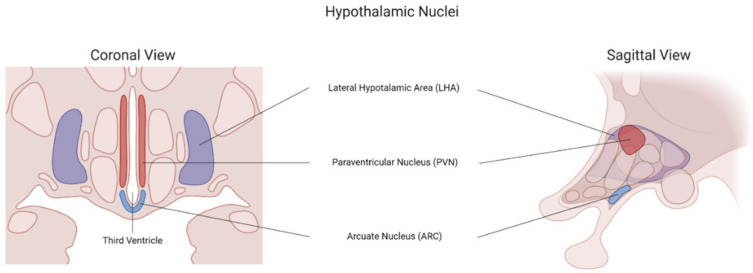
Hypothalamic nuclei in food intake regulation. Coronal and sagittal views.

The hypothalamic nuclei act as the front door where peripheral metabolic signals are first sensed and translated into orexigenic and anorexigenic signals to regulate energy balance (Figure 2). Surrounding the ventral part of the third ventricle lies the ARC, where a semipermeable BBB formed by a highly fenestrated local capillary network allows peripheral signals to enter the CNS [34]. The POMC/CART-expressing neurons represent the anorexigenic population of the ARC; however, they are also present in the NST. On the contrary, the AgRP/NPY neurons make up the orexigenic population of the ARC. The POMC/CART and the AgRP/NPY neurons co-express receptors and share similar efferent connections to the forebrain despite being functionally opposite to each other [93]. In the anterior lobe of the pituitary gland, POMC is cleaved into adrenocorticotropin hormone (ACTH), β-lipotropic hormone (β-LPH), and *N*-POMC; however, in the ARC, ACTH and β-LPH are further cleaved to yield α and β melanocyte-stimulating hormones (α-MSH, β-MSH), respectively [94]. α-MSH is secreted in response to leptin and other homeostatic peripheral signals such as GLP-1 and PYY. AgRP/NPY neurons are activated by ghrelin and inhibited by PYY and leptin; however, they are also stimulated by anticipatory sensorial food cues and appear to remain activated long after the stimuli have been removed [95,96]. Activation of AgRP/NPY neurons increases food intake, and rodent models have shown that hunger is interpreted as an aversive feeling. Thus, it has been hypothesized that the rewarding effect behind food intake comes from the removal of this aversive sensation of hunger, thus becoming a reinforcing experience [97]. In humans, the density of AgRP/NPY neurons is negatively correlated with BMI [98].

The PVN is located dorsal to the ARC, which represents the downstream hypothalamic structure in the leptin–melanocortin pathway. The neurons in the PVN express the single-minded 1 transcription factor (*SIM1*), a factor essential for the neurogenesis of the structures, and genetic variants in the *SIM1* have been associated with severe early-onset obesity [99]. This neuronal population contains the anorexigenic MC4R, expressing neurons that respond to α-MSH secreted upstream in the ARC-POMC/CART neurons. Mutation in the MC4R is one of the most common causes of non-syndromic monogenic obesity in humans, with a prevalence of around 4% [26,100]. AgRP and NPY inhibit MC4R neurons. The α-MSH analog setmelanotide is approved for some homozygous monogenic mutations (LEPR, MC4R, POMC, PCSK1) in the leptin–melanocortin pathway to treat severe obesity and is being studied for heterozygous mutations as well [101,102]. Stimulation of the MC4R by POMC/CART neurons regulates energy balance through a decrease in food intake and effects in energy expenditure, cardiovascular functions, and glucose homeostasis [103]. In another fMRI study, patients with obesity and MC4R deficiency had a high striatal (dorsal and ventral striatum) activity to palatable foods in the sated state compared with non-carrier with obesity. This finding suggests that the integrity of the leptin-MC4R pathway is needed for the hyporesponsiveness observed in non-carriers with obesity [104].

#### 3.3.2. The Lateral Hypothalamic Hypocretin Pathway

The lateral hypothalamic area (LHA) is less studied than the ARC and PVN. LHA’s orexigenic effects are mediated by the hypocretins, namely, the melanin-concentrating hormone (MCH) and orexins [105]. The neurons that secrete these hypocretins are intermingled and share similar connections with other brain structures [106,107]. MCH and orexin neurons project to the brainstem and the reticular activating system [108]. Projections to autonomic structures in the medulla and spinal cord aid in the regulation of salivation, gastric motility, and pancreatic hormone secretion [109]. Additionally, the hypocretin system has a connection with the monoaminergic arousal system [107]. Interestingly, the NAc, which is crucial in the reward system, only receives input from MCH neurons [110]. The monoaminergic system represents the connection between the LHA and the reward system through neurotransmitters such as 5-HT and DA. The areas to where hypocretins project can serve as a framework to better understand their effect on food intake regulation.

To date, few neuroimaging studies have assessed the hypothalamus. Its size, nuclei sub-division, and anatomical location make it a challenging structure to investigate. Page and colleagues observed a decrease in hypothalamic CBF after glucose but not after fructose ingestion [111]. Additionally, only glucose ingestion caused deactivation in the striatum and increased connectivity between the hypothalamus and the striatum. Another study looked at the responses in several brain areas, including the hypothalamus, after two days of overfeeding. The observations showed reduced activation in the hypothalamus (and visual areas) compared with a eucaloric diet [112]. Other studies have shown an attenuated hypothalamic response in patients with obesity compared with lean patients after glucose ingestion (either oral or intragastric) [68,113]. Contreras-Rodríguez et al. studied the difference in connectivity between the medial and LHA with some reward regions in patients with obesity and normal weight. They observed increased connectivity between the medial hypothalamus and reward areas such as the striatum and the ACC and a decreased connectivity with the middle frontal gyrus in patients with obesity [114].

In summary, the hypothalamus responds to peripheral metabolic signals. The ARC acts as the main door where the metabolic feedback is decoded into hunger (by AgRP/NPY neurons) or satiety/satiation (by POMC/CART neurons) signals and projected to the PVN. From the PVN, these metabolic signals modulate the activity of the brain areas involved in reward, aversion, memory, learning, and decision making to regulate eating behavior. Hypocretins—from the LHA—represent an important link between the homeostatic and hedonic systems and strongly modulate reward-related brain areas.

#### 3.3.3. Sensitive and Sensorial Information

Information carried by some cranial nerves plays an essential role in regulating energy intake [115]. The sensorial information of food has both a conscious and unconscious component. Not only that, some of this sensorial information reaches the cerebral cortex without intermediate stops within the CNS and can easily influence the hedonic system and override homeostatic regulation.

In the mouth, somatosensory information from foods travels through the trigeminal nerve to provide the organism information such as temperature, consistency, and texture [116]. The facial, glossopharyngeal, and vagus nerves decode gustatory information from the four basic tastes (i.e., sweet, sour, salty, bitter) and umami (MSG). These taste modalities are critical for food selection and preference and serve as an innate detector to characterize nutrients as acceptable, potentially toxic, and/or essential [117]. Taste is also one of the most important components of hedonic eating as it transforms—through reward—an unexciting vital process, such as energy intake, into a liked and wanted experience. Taste perception also has a particular cultural influence [118,119]. Not only that, fMRI studies have shown how there is inter-individual variability and overlap in areas that process the four basic tastes and MSG [120]. In the NST, gustatory information is translated and projected to higher CNS structures that control hedonic eating and hunger [121]. Visceral and gustatory feedback from the NST is then projected to the parabrachial nucleus (PBN) and the thalamus, where taste and metabolic status information is sensed. Additionally, the PBN is critical in developing and retaining conditioned taste aversions, which is the opposite of reward in learning processes in eating behavior [117]. In rodents, stimulating calcitonin gene-related peptide (CGRP) expressing neurons in the lateral external PBN inhibits food intake, while its inactivation leads to increased food consumption [122]. Some fMRI studies have shown that sweet taste activates reward areas, suggesting that sweet taste has a higher hedonic value than the other basic tastes or MSG [123,124]. Moreover, other fMRI studies have shown that branding and cost can also influence taste and reward perception of identical foods [59,125,126,127].

The sense of sight is also essential in food intake, as it allows the organism to identify food resources as palatable, safe, noxious, and/or nutritive based on previously learned experiences through sensorial and metabolic information decoded by the CNS. Visual information is the most tested modality in neuroimaging studies. In an fMRI study, visual food cues of high-energy foods produced a higher response in reward-related brain areas in the morning compared with other times of the day [128]. Another study demonstrated a positive correlation between BMI and the degree of activation in the dorsal striatum, anterior insula, claustrum, posterior cingulate, and the postcentral and lateral OFC in response to visual food cues [129].

The sense of smell is also the only dual sense, constructed from both external (orthonasal) and internal (retronasal) modalities [130]. Smell and other senses such as taste and sight are consciously perceived and closely linked with unconscious reward, learning, and memory systems in the CNS [131]. Smell and taste—and perhaps other senses—encompass the spectrum known as flavor. Using BOLD-MRI, Small et al. studied the brain responses to flavor by delivering congruent and incongruent olfactory plus gustatory stimuli (i.e., vanilla odor plus sweet (congruent) or salty (incongruent) stimuli). They found that the insula, OFC, and ACC are essential for perceiving the flavor composite [132].

In summary, pre-ingestive and post-ingestive sensitive and sensorial stimuli appear to be a potent modulator in food intake that constitutes fast and continuous feedback to the hedonic system. This sensitive/sensorial feedback is dynamically modulated throughout the eating phase by peripheral metabolic signals from the self-regulatory system (i.e., appetition). Importantly, these sensorial reward-related signals from food (i.e., taste, smell, temperature, consistency, visual appeal) can easily override homeostatic regulation.

### 3.4. Stages of Food Intake: Hunger, Satiety, and Satiation

Few neuroimaging studies have assessed the neural correlates of each stage of food intake (i.e., hunger, satiation, and satiety). The continuum of processes that orchestrate food demand has elaborate study designs to assess each stage individually. Moreover, terminology in food intake still requires standardization and consensus (Table 1) as some definitions can often be used interchangeably by some authors. Some studies have investigated brain activity after reaching satiation with liquid meal stimuli [133]. Thomas and colleagues observed decreased BOLD-fMRI activity in the vmPFC, OFC, NAc, hypothalamus, and insula and increased activity in the dorsolateral PFC after reaching satiation with an ad libitum liquid meal. Other neuroimaging studies have observed sex-specific responses to satiation in reward-related brain areas [134,135].

## 4. Hedonic Food Intake Regulation

**Figure 3:**
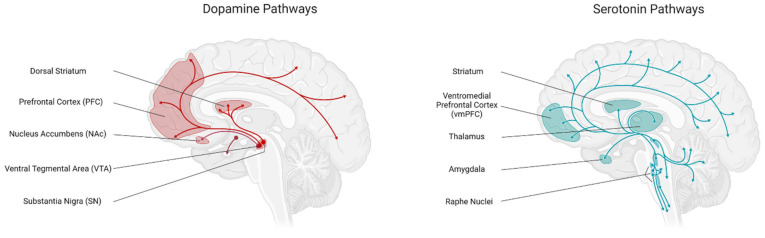
Central serotonin and dopamine pathways in food intake regulation.

In the mid-1900s, Olds and colleagues carried out an experiment that set a precedent for the anatomical correlates for reward in the CNS. They applied electric currents in different rat brain regions after pressing a lever [136]. In some regions, the effect was so potent that the rodents stopped other vital processes such as feeding and pressed the lever repeatedly to reproduce this effect. This observed behavior of exploring, learning, and repeating to obtain an expected or desired response established an anatomical correlate for reward processing in the CNS. Later, other studies unveiled the link between these “reward“ brain regions with dopaminergic neuronal populations, especially in the midbrain [137]. This midbrain dopaminergic input strongly influences structures such as the striatum, the amygdala, and the prefrontal and orbitofrontal cortices. Additionally, the connection between neurotransmitters (such as DA and 5-HT) with these reward brain structures plays a key role in the hedonic regulation of food intake (Figure 3) [138,139,140]. Dopaminergic pathways are also critical to code for reward prediction errors and are essential in learning and decision-making [141].

Hedonic eating can be defined as eating merely to produce pleasurable feelings, disregarding the energy status [5]. While the primary purpose of food is to provide the energy needed to survive and thrive, hedonic and homeostatic regulation is closely tied within the brain for specific reasons. The perception of food valence is one of the essential attributed features to food regarding hedonic eating. It is built based on physical characteristics decoded by our senses (such as sight, taste, and smell) and shaped by personal socio-cultural, economic, and nutritional information that, altogether, carves our perception of reward [59,125,126,127,142,143]. This complex construct provides food with “liking” and “wanting” features [5]. “Wanting” can be explained by incentive salience. Incentive salience is a non-physical construct from previously learned reward-predicting values. The neural circuitries behind “wanting” are mainly dopaminergic pathways. On the other hand, “liking” relies on smaller cannabinoid and opioid pathways [144,145,146,147]. The overlap of these two circuitries has led to the development of neurocognitive theories to explain food intake dysregulation in obesity [5,142,143,144,145,146,148,149,150,151,152,153,154]. The neurobiological resemblance of circuitries involved in food-reward with circuitries involved in substance-abuse disorders has led to interrogate whether the dysregulation of food intake in obesity fits into an addiction model (i.e., food addiction disorder) [155,156,157,158,159,160]. However, this proposal has received contradictory and non-conclusive opinions [161,162].

Neurotransmitters and neuropeptides such as DA, 5-HT, opioids, and cannabinoids are mostly responsible for the hedonic regulation in food intake. These neurotransmitters make numerous reciprocal connections between reward-related brain structures and serve as second messengers to the CNS to translate peripheral metabolic signals [38].

### 4.1. Neurotransmitters and Neuropeptides

#### 4.1.1. Dopamine

DA is one of the most studied monoaminergic neurotransmitters in food intake regulation. The mesolimbic pathway projects from the VTA in the midbrain to the striatum, both the ventral (consisting of the NAc and the olfactory tubercule) and dorsal (consisting of the caudate nucleus and putamen) striatum, and composes the main dopaminergic pathway in food intake regulation. However, other DA projections are directed to cortical regions such as the OFC and PFC and limbic regions such as the insula, hippocampus, and amygdala. Even with the broad involvement in hedonic eating regulation, DA’s role is often oversimplified as the only neurotransmitter involved in reward regulation [163,164]. Brain processes involving DA—and 5-HT—are subject to habituation soon after repetition. This reward habituation in food intake causes a seeking behavior to re-experience reward through new food stimuli [165]. Food reinforcement (through reward processes) and habituation are key determinants of eating behavior and important predictors of the degree and variance of caloric intake (and obesity) [165,166,167]. Notwithstanding this habituation, rewarding DA stimuli are decoded into anticipatory cues (e.g., the sight, smell, or thought of food) that predict reward, becoming conditioned behaviors. Certain foods, especially those with high sugar and fat content, can trigger addictive-like behaviors [168,169,170], but, as mentioned before, the rewarding properties of a specific type of food depend on its palatability and external factors such as availability, economics, socio-cultural context, visual appeal, and incentives.

Furthermore, peripheral metabolic signals also modulate the rewarding perception of food by signaling to the mesolimbic and mesoaccumbens DA pathways or through indirect signaling to the hypothalamus and then projecting to the VTA [171,172,173,174,175,176,177,178]. DA’s role in overeating and obesity has been observed in patients with the Taq1A polymorphism, where A1 carriers have reduced D2R availability in the CNS [179]. fMRI studies observed increased striatal activity in response to food cues in A1 allele carriers with obesity but not in lean carriers. This finding suggests that this mutation leads to increased sensitivity to food cues and overeating [179]. Another study assessed dopamine release in response to palatable food (i.e., milkshake) intake using a combination of fMRI and PET with 220–370 MBq [^11^C]raclopride [180]. An immediate DA release in the NST, the lateral ventral anterior nucleus of the thalamus, and the frontal operculum/anterior insular cortex was observed after milkshake consumption. This immediate DA release with sensorial stimuli is consistent with previously mentioned findings in rodents from Chen et al., who found that AgRP neurons are stimulated before food consumption [95]. Interestingly, a delayed (i.e., 15–20 min) post-meal DA release in ventral posterior medial thalamus, insular cortex, and basolateral amygdala was also observed [180]. These observations provide an interesting framework on how peripheral metabolic feedback is sensed by the CNS and then reinforced by the DA reward system in the brain to aid learning and decision-making in food intake [181].

In summary, DA is an essential—but not the only—signal in hedonic food intake regulation, strongly shaping eating behavior through cognitive and conditioned learning processes.

#### 4.1.2. Serotonin (5-HT)

5-HT is a biogenic amine that is synthesized in the central and the enteric nervous system. Caudal projections are sent from populations B1–B4 in the raphe nuclei to the cerebellum, midbrain, pons, medulla, and spinal cord, whereas ascending projections from B5–B9 are projected forebrain structures such as the cerebral cortex, striatum, amygdala, and hypothalamus [144]. 5-HT is associated with mood, cognitive, autonomic, and homeostatic regulation, and its levels are inversely correlated with food intake [144]. Recent studies suggest that serotonin-induced hypophagia acts via downstream activation of the leptin–melanocortin pathway [182].

Most of the neuroimaging studies investigating the role of 5-HT have been performed in patients with eating disorders (e.g., anorexia nervosa and bulimia nervosa) using mainly PET and SPECT with 5-HT radioligands [183,184,185,186]. In a study using PET in patients with obesity, Haahr and colleagues found a strong association between BMI and serotonin receptor 4 (5-HT_4_R) density in the NAc and the ventral pallidum, suggesting an upregulation of 5-HT_4_R in these areas [187]. Other fMRI studies have explored the role of 5-HT and reward prediction, showing that 5-HT activity in the ventral and dorsal striatum correlated with reward prediction at short- and long-time scales, respectively [188,189]. With these findings, the authors hypothesized that 5-HT activity in the striatum controls the time scale of reward prediction. In another study, activation of 5-HT_2C_R with the agonist meta-chlorophenylpiperazine demonstrated decreased BOLD-fMRI activity in response to visual food cues in reward-associated brain areas. Additionally, consumption of palatable food and appetite decreased, and satiation increased [139,190]. The 5-HT_2C_R agonist Lorcaserin (withdrawn by the FDA in February 2019) demonstrated decreased BOLD activity to visual palatable food cues (high-calorie or high-fat pictures of cakes, onion rings, and similar foods) in the parietal and visual cortices [191]. To date, pharmacotherapy for eating disorders is limited to just a handful of medications that, while well-tolerated, cause a wide variety of side effects [192]. While this provides a glance into the role of some neurotransmitters in eating disorders, further studies are required to understand and develop more effective and safer treatments for patients with eating disorders.

The available evidence suggests that 5-HT has pleiotropic effects on mood and cognition that extend to modulation of the hedonic properties of food through the agonism of some of its receptors.

#### 4.1.3. Endocannabinoid and Opioid Systems

As stated before, the endocannabinoid and opioid circuitries are responsible for the “liking” process in food intake [193,194]. The most notable endocannabinoids, anandamide (also known as *N*-arachidonoyl ethanolamine) and 2-arachidonoylglycerol (2-AG), are synthesized from lipid derivates of arachidonic acid and act on the CB1 receptor (CB1R). Rodents lacking diacylglycerol lipase isoform α (DGL-α; an enzyme involved in the biotransformation of 2-AG from diacylglycerol) display decreased food intake and a lean phenotype, suggesting a potential clinical application of DGL-α inhibition in patients with obesity [194]. However, other endocannabinoid-like molecules, such as oleoylethanolamide (OEA), also have an important role in food intake regulation, particularly in satiation [193]. In rodent studies, OEA (an N-acylethanolamine derived molecule) has been proposed as a satiation signal by activating intestinal PPAR-α receptors that stimulate afferent vagal fibers to induce meal termination through an NTS-PVN pathway [193,195,196]. Additionally, OEA has been proposed as a key connection between the hedonic and homeostatic systems, with potential clinical implications for the treatment of obesity and non-alcoholic fatty liver disease [197,198,199]. Together with the endocannabinoids, endocannabinoid-like molecules and their receptors and enzymes constitute the expanded endocannabinoid system or endocannabinoidome [200]. The endocannabinoidome has been proposed to be influenced by dietary fatty acids, to impact the microbiome, and to influence food intake regulation through these interactions [201,202]. The CB1R is responsible for signaling the homeostatic and hedonic regulation of food intake and energy expenditure by endocannabinoids [203]. The CB1R is widely expressed throughout the CNS in regions such as the VMH, PVN, LHA, medial preoptic area, NST, AP, and the ARC [204] and has been directly correlated with BMI [205]. It has also been observed that food deprivation and obesity elevate endocannabinoid levels in the hypothalamus [206,207]. The role of endocannabinoids in food intake regulation has led to the development of anti-obesity medications such as the non-neutral antagonist Rimonabant. Despite its success in weight loss, it was removed from the market in 2007 by the FDA due to the increased risk of depressive disorders and suicidality during post-marketing surveillance [208]. In an fMRI study performed by Horder and colleagues, administrating 20 mg of Rimonabant for seven days inhibited rewarding neural activity responses to food cues (both visual and gustatory) in the ventral striatum and OFC [209]. Another fMRI study observed increased activity in response to chocolate stimuli in the midbrain ACC, caudate, and putamen after administration of the neutral CB1 antagonist tetrahydrocannabivarin [210].

The endogenous opioid circuitry in the CNS is built up by β-endorphins, enkephalins, and dynorphins as well as the opioid receptors μ, δ, and κ [211]. The μ opioid receptor (MOR) is strongly implicated in food intake regulation, predominantly in reward and the incentive motivational value of foods and food-related cues [212]. MOR availability in brain reward areas was assessed with PET scans using [^11^C] carfentanil (MOR ligand) during rest and exercise situations; brain activity in response to palatable (e.g., cookies and pizza) and non-palatable (e.g., lentils and bread) food cues was assessed with BOLD-fMRI. Palatable foods activated areas such as the amygdala, ventral striatum, and hypothalamus. MOR availability was negatively correlated with the reward response in fMRI scans [213]. The formulation of naltrexone extended-release (ER) plus bupropion-ER (brand name Contrave) was approved in 2014 by the FDA for long-term weight management of obesity. Bupropion is a dopamine/norepinephrine reuptake inhibitor, and naltrexone is an opioid receptor antagonist [214]. Some studies have evaluated the effect of Contrave in response to food cues using neuroimaging, consistently demonstrating increased activation in brain areas involved in inhibitory control and saliency attribution, interoception, and memory, showing a decreased local functional connectivity density in the medial PFC, which is associated with cravings [158,215]. Murray et al. examined the effects of naltrexone on a set of visual pleasant (chocolate in the mouth with a picture of chocolate) and unpleasant (strawberry flavored medicine in the mouth with a picture of molded strawberries) food stimuli. The investigators observed that naltrexone decreased activation in the dorsal ACC and caudate nucleus in response to the pleasant food stimulus and increased activation in the amygdala and anterior insula to unpleasant food [216].

In summary, the endocannabinoid system—through its CB1R—acts as a potent food intake regulator by increasing appetite, albeit with important effects on mood. Additionally, endocannabinoid-like molecules display an opposite (i.e., orexigenic) effect on food intake regulation. The extended endocannabinoid system appears to have a significant effect on regulating food intake through effects on the gut, diet, and microbiome.

### 4.2. Homeostatic and Hedonic System Overlap and Interaction

**Figure 4:**
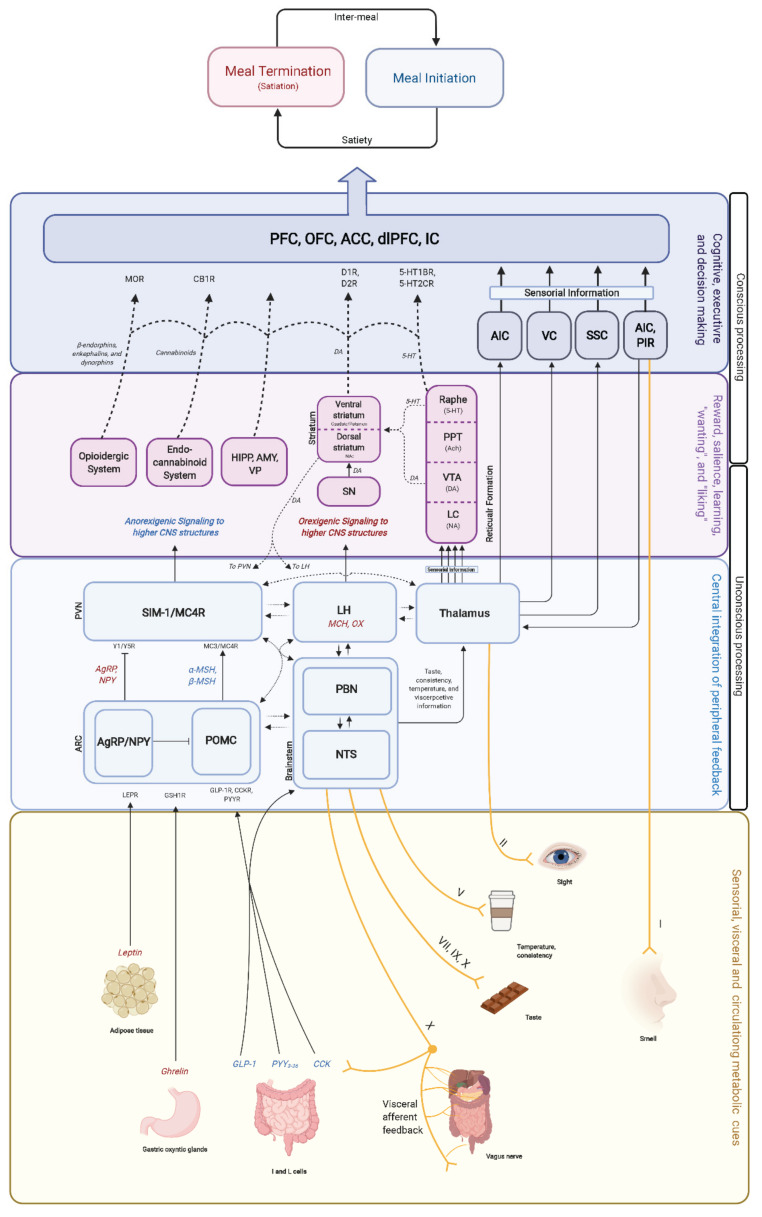
Food intake is regulated by internal and external stimuli perceived through conscious and unconscious pathways in a multi-level fashion. Internal and external signals (yellow bottom box) are transmitted through hormonal and neural pathways to the CNS (solid and dashed lines). Orexigenic (red letters) and anorexigenic (blue letters) hormonal feedback from the periphery (gut and adipose tissue) is projected and unconsciously sensed by the hypothalamus and brainstem (light blue boxes). Sensorial and visceroceptive information afferents (solid yellow lines) are perceived consciously by the CNS. From the hypothalamus and thalamus, information is projected to reward-related brain areas (purple boxes), where a circuitry of neurotransmitters such as 5-HT, dopamine, and endorphins are projected to higher cognitive brain areas to regulate eating behavior (dark blue boxes). PFC, prefrontal cortex; OFC, orbitofrontal cortex; ACC, anterior cingulate cortex; dlPFC, dorsolateral prefrontal cortex; MOR, mu opioid receptor; CB1R, cannabinoid receptor 1; D1R, dopamine receptor 1; D2R, dopamine receptor 2; DA, dopamine 5-HT1BR serotonin 1B receptor; 5-HT2CR, serotonin 2C receptor; 5-HT, serotonin (5-hydroxytryptamine); AIC, agranular insular cortex; VC, visual cortex; SSC, somatosensorial cortex; PIR, piriform cortex; PVN, paraventricular nucleus; SIM-1, single minded 1 gene; MC4R, melanocortin receptor 4; LH, lateral hypothalamic area; MCH, melanocortins; OX, orexins; Y1R, neuropeptide Y receptor 1; Y5R, neuropeptide Y receptor 5; MC3R, melanocortin 3 receptor; AgRP, Agouti-related peptide; NPY, neuropeptide Y; a-MSH, alpha melanocyte stimulating hormone; b-MSH, beta melanocyte stimulating hormone; ARC, arcuate nucleus; POMC, pro-opiomelanocortin; PBN, parabrachial nucleus; NTS, nucleus tractus solitarius/nucleus of the solitary tract; LEPR, leptin receptor; GSH1R, growth hormone stimulant receptor 1; GLP-1R, glucagon-like peptide 1 receptor; CCKR, cholecystokinin receptor; PYYR, peptide YY receptor; GLP-1, glucagon-like peptide 1; CCK, cholecystokinin; PYY, peptide YY; X, vagus nerve; VII, facial nerve; IX, glossopharyngeal nerve; V, trigeminal nerve; II, optic nerve; I, olfactory nerve.

In humans, the regulation of food intake by the homeostatic and hedonic systems is a complex process (Figure 4), with some structures or hormones appearing to have a single role in this regulation, while others appear to be always involved and responsive to any stimulus. However, the complexity of this regulation begets complexity to its study, and these findings might be related to our current methodological and technological limitations. Previously, the homeostatic system was thought to be independent of its hedonic counterpart, and while it operates under unconscious control merely driven by the individual’s metabolic status, it is easily overridden by strong reward and motivational processes from the hedonic system. As we previously discussed, this complex crosstalk and strong bidirectional influence between systems are essential for survival. Unfortunately, the hedonic system has been particularly vulnerable to the modern food environment, and the self-regulatory (i.e., homeostatic) system has not been able to compensate for these phenomena [217].

## 5. Future Directives in Food Intake Neuroimaging

Even with the outstanding improvements in technology over the last 20 years, the field of neuroscience still lacks practical, non-invasive tools to study the human brain. The complexity of neural structures and their connections represent a challenge that demands the development of new sophisticated tools as well as a deeper understanding of the underlying neural substrate in order to develop a suitable study methodology to measure this complexity [218].

Current state-of-the-art neuroimaging techniques are limited in their ability to measure the biological processes related to hunger, satiation, and satiety. Unfortunately, one must acknowledge that defining the circuitry of the pathways inside the CNS is extremely difficult using cerebral blood flow measurements (MRI) or sugar uptake (PET/SPECT). For example, these techniques cannot differentiate between excitatory or inhibitory neuronal activity, identify the neurotransmitter(s) in play, or determine a stimulus’s direction(s).

All current human neuroimaging techniques are limited by spatial resolution, on the order of 0.8–3 mm per voxel for MRI and 2–5 mm for PET/SPECT, vastly larger than individual neurons (0.01–0.02 mm) or even cortical columns (0.3–0.6 mm). PET and SPECT measure (indirectly and directly, respectively) the γ rays emitted from molecules tagged with positron-emitting isotopes. Cerebral blood flow and metabolism can be assessed by measuring the distribution of these isotopes [219]. SPECT gives a lower spatial resolution than PET, and both are costly and expose the patient to radiation [220].

Furthermore, there is a critical need to standardize the methodologies of neuroimaging studies in food intake to enhance the external validity of their findings and interpretations. Researchers should follow the latest available recommendations when developing study designs [221]. While the complexity of eating behavior still represents a major limitation, standardizing the design, analysis, and interpretation of food intake neuroimaging studies will strengthen the clinical applicability of study findings. Factors such as age, sex, weight status, study design, and interpersonal variability in response to stimuli need to be considered in all such studies. This approach will help us to develop imaging protocols specific to certain conditions, leading to a more individualized approach to understanding, diagnosing, and treating conditions that arise from dysregulation in food intake.

Neuroimaging tool development for mapping the hedonic and homeostatic components of food intake regulation is an exciting area of research for both human and animal studies. The novel applications of tools such as electroencephalography, magnetoencephalography, and functional near-infrared spectroscopy will enhance our understanding of food intake regulation in humans. Additionally, obesity-related mutations in food intake regulation pathways and the development of new anti-obesity medications that target these pathways represent an interesting area of focus where neuroimaging, in conjunction with genomics and proteomics, could help to determine different obesity phenotypes and individualized responses to therapy.

## 6. Conclusions

Neuroimaging studies are unveiling an exciting landscape of the crosstalk between the hedonic and homeostatic systems, contrary to the former statement of a parallel, independent regulation. Moreover, understanding that the nutritive properties of food unconsciously modulate the reward systems in the brain through gut hormones reveals the role of reward processes in the brain that regulate an essential process in all living beings’ energy intake. Despite the outstanding advances of human neuroimaging, the complexity of the neural components, stages, and systems regulating food intake demand more sophisticated study designs to assess these variables in unification and in isolation to understand and diagram this intricate regulation thoroughly. Further innovation in neuroimaging techniques and the development of sophisticated technology to assess neural activity and connections in humans is needed to overcome the innate complex limitations of food intake physiology. Understanding the composite network that regulates food intake will allow scientists to develop strategies to prevent, explain, and treat conditions caused by dysregulation in food intake.

## Figures and Tables

**Table 1:** Definitions in homeostatic and hedonic food intake regulation.

Definitions	
Appetite	Desire to fulfill energetic needs, divided into hunger, satiety, and satiation
Hunger	Drive to consume
Satiation	The process that brings an eating episode to an end (intra-meal inhibition)
Satiety	The process that inhibits eating or hunger in the postprandial period (inter-meal inhibition)
Fullness	The visceral sensation of gastric distention
Reward	The stimulus for which animals are willing to work for
Hedonism	Derived from the Greek hēdonikós, “pleasurable”; a sensation of positive feelings
Hedonic Eating	Eating solely to elicit pleasurable feelings and/or to escape from anhedonic states, disregarding the metabolic status or the nutritional value of the food consumed.
Appetition	The post-oral process where nutrient properties of food promote intake beyond inhibition processes (e.g., satiety and satiation).
Preference	Selection among different options based on subjective liking
Incentive Salience	The cognitive process of “wanting” a rewarding stimulus based on its attributed salient characteristics
Reward prediction error	Difference between the received and predicted reward from a stimulus

## Data Availability

Not applicable.
